# Assessment of Entrepreneurial Orientation in Vocational Training Students: Development of a New Scale and Relationships With Self-Efficacy and Personal Initiative

**DOI:** 10.3389/fpsyg.2019.01125

**Published:** 2019-05-14

**Authors:** Arantxa Gorostiaga, Jone Aliri, Imanol Ulacia, Goretti Soroa, Nekane Balluerka, Aitor Aritzeta, Alexander Muela

**Affiliations:** ^1^Department of Social Psychology and Behavioral Sciences Methods, University of the Basque Country UPV/EHU, San Sebastian, Spain; ^2^Department of Personality, Assessment and Psychological Treatment, University of the Basque Country UPV/EHU, San Sebastian, Spain; ^3^Department of Basic Psychological Processes and Development, University of the Basque Country UPV/EHU, San Sebastian, Spain

**Keywords:** entrepreneurial orientation, self-efficacy, personal initiative, measurement invariance, multi-group confirmatory factor analysis

## Abstract

Having emerged as an important concept in the organizational field, entrepreneurial orientation has also become a key idea in the context of education. Indeed, entrepreneurial education is now one of the common objectives for education and training systems in the European Union. Despite its importance, however, there is a scarcity of valid and reliable measures for assessing entrepreneurial orientation in students. The present study aimed to address this by developing and examining the psychometric properties of the Entrepreneurial Orientation Scale (EOS). A second objective is to study the relationships between entrepreneurial orientation and gender, self-efficacy, and personal initiative. The sample comprised 411 vocational training students (50.36% male, 49.64% female). The final version of the instrument comprised 32 items assessing six dimensions: innovativeness, risk-taking, proactiveness, competitiveness, achievement orientation, and learning orientation. The EOS showed good psychometric properties and its dimensions demonstrated concurrent relationships with self-efficacy and personal initiative. The EOS may be used to measure entrepreneurial orientation in the educational context and to evaluate interventions designed to promote an entrepreneurial spirit in schools, colleges, and universities.

## Introduction

Since the 1980s, increasing importance has been attached to the concept of entrepreneurial orientation (EO) (Miller, 1983; Covin and Slevin, 1989), especially in the literature on entrepreneurship and organizational performance. Various studies have sought to define this concept in terms of certain psychological, sociodemographic, and entrepreneurial profiles (Shapero and Sokol, 1982; Lumpkin and Dess, 1996; Veciana, 1999; Krauss et al., 2005; Rauch et al., 2009; Vij and Bedi, 2012). For example, Lumpkin and Dess (1996) define EO as the processes through which organizations seek to develop a strategic basis for decisions and entrepreneurial actions. Krauss et al. (2005) emphasize the psychological nature of EO and point out that orientations, in contrast to traits, are culturally determined and influenced by context.

The first dimensions of EO to be consistently identified by organizational research were innovativeness, risk-taking, and proactiveness (Covin and Slevin, 1991). In the organizational context, innovativeness refers to the propensity toward creativity and experimentation through the introduction of new products and services, as well as to technological leadership in new processes. Risk-taking is the degree to which firms or managers are willing to consider investing in and committing resources to projects that may well fail, and to assume the risks associated with such initiatives. Finally, proactiveness is about seeking opportunities and refers to how an organization goes about anticipating future market needs. Lumpkin and Dess (1996) subsequently proposed another two dimensions of EO: competitive aggressiveness and autonomy. Competitive aggressiveness refers to the intensity of approach and head-to-head posturing that a company may need in order to compete with its rivals. The autonomy dimension reflects the independent and autonomous actions that are implemented by leaders and teams with the aim of launching a new venture. Krauss et al. (2005) later added two more elements to this framework, namely *achievement orientation* and *learning orientation*. Firms or individuals with a strong achievement orientation perform better on non-routine tasks and take responsibility for their performance. Learning orientation refers to the ability to learn from both positive and negative experiences and to the willingness to question assumptions or mental models in the pursuit of success.

Several studies have suggested that the different dimensions of EO are intercorrelated (Bhuian et al., 2005; Tan and Tan, 2005), or even that they may be subsumed under a single factor (Covin et al., 1994; Wiklund and Shepherd, 2003). However, other authors consider them to be independent aspects of a multidimensional construct (Lumpkin and Dess, 1996; George, 2011). In the meta-analysis carried out by Rauch et al. (2009), 37 of the 51 studies reviewed considered the EO construct to be unidimensional, while the remainder viewed it as multidimensional. The debate over the dimensionality of the construct therefore remains open.

Although the notion of EO emerged in the organizational context, it is now a key concept in the field of education, especially in the sphere of vocational training. This is illustrated by the fact that a “sense of initiative and entrepreneurship” is regarded by the European Commission as one of the key competences for lifelong learning (European Commission, 2007). Likewise, entrepreneurial education is one of the three key areas targeted by the Entrepreneurship 2020 Action Plan (“Promoting the spirit of entrepreneurship in schools and universities”), which the European Commission adopted in January 2013.

When the aim is to study entrepreneurial orientation in contexts other than the organizational one (e.g., the educational context), the focus needs to be on teaching and learning activities, as well as on other everyday activities. This has been done, for example, by Bolton and Lane (2011) with university students, and Kurniawan et al. (2019) with high school students.

Thus, in the present study, and drawing on existing models, we define entrepreneurial orientation as the psychological propensity of individuals to propose innovative and creative solutions to problems and to show proactiveness, autonomy, and competitiveness in the various spheres of their life, assuming the risks associated with their decisions and showing a marked orientation toward achievement and learning. Consequently, we take as our reference the seven dimensions of entrepreneurial orientation considered by Krauss et al. (2005) and apply them to a context other than the organizational one.

Research on gender differences in EO and its dimensions has yielded inconsistent results. Some authors have reported a higher level of EO among men (Bilić et al., 2011; Goktan and Gupta, 2015), although a study involving undergraduates found no such difference (Hunt, 2016). As regards the dimensions of EO, some studies have found that men score higher on innovativeness (Ayub et al., 2013; Reyes et al., 2014). However, Pérez-Quintana (2013) found no difference between men and women in this respect, and in the multi-country study by Lim and Envick (2013) a gender difference was observed in Fiji but not in the United States, Korea, or Malaysia. With regard to risk-taking, most studies have found higher scores among men (Ayub et al., 2013; Lim and Envick, 2013, in three of the four countries studied; Taatila and Down, 2012; Pérez-Quintana, 2013). However, Reyes et al. (2014) found no gender differences on the dimension which they labeled “risk propensity.” For the proactiveness dimension, some studies report higher scores in women (Ayub et al., 2013; Marques et al., 2018), while others associate higher scores with men (Callaghan and Venter, 2011; Taatila and Down, 2012; Pérez-Quintana, 2013). Finally, men are generally reported to score higher on competitive aggressiveness and autonomy (Ayub et al., 2013; Lim and Envick, 2013). Given these inconsistent results regarding the relationship between gender and EO, investigating possible differences in the educational field could make a useful contribution.

Several studies have analyzed the relationship between EO and a series of variables in the literature on entrepreneurship, including self-efficacy and personal initiative. The study of these two variables is particularly relevant because there is evidence that individuals choose to become entrepreneurs most directly because they are high in self-efficacy (Zhao et al., 2005), while recent research has underlined the positive and significant association between personal initiative and social entrepreneurial behavior (Nsereko et al., 2018).

Self-efficacy is a concept that describes an individual’s belief in his/her ability to succeed in a given task, and it could explain human behavior, since it plays an influential role in determining an individual’s choice, level of effort, and perseverance in meeting certain objectives (Bandura, 1977; Chen et al., 2004; Sesen, 2013). In the scientific literature on entrepreneurship, researchers have tended to study the construct of entrepreneurial self-efficacy (ESE) as a key antecedent of new venture intentions (Boyd and Vozikis, 1994). However, as McGee et al. (2009) point out, disagreement exists as to whether the ESE construct is more appropriate than general self-efficacy (GSE) for that purpose. In this respect, some studies have found that self-efficacy is positively related to EO (Hashemi et al., 2012; Arrighetti et al., 2013; Malebana and Swanepoel, 2014; Mohd et al., 2014) and that entrepreneurs score higher on self-efficacy than do non-entrepreneurs (Markman et al., 2005).

Personal initiative is defined as a set of behaviors related to proactiveness, persistence, and self-starting, which are necessary when people encounter difficulties in achieving goals (Frese and Fay, 2001). Some studies have concluded that entrepreneurs show higher levels of personal initiative than do non-entrepreneurs (Frese et al., 1997; Frese and Fay, 2001; Lisbona and Frese, 2012). Furthermore, personal initiative shows positive correlations with entrepreneurial success (Crant, 1995; Koop et al., 2000; Korunka et al., 2003; Krauss et al., 2005) and with entrepreneurial orientation (Koop et al., 2000; Krauss et al., 2005). However, these relationships have not been widely studied outside the organizational field, and more research is therefore needed.

Although instruments for assessing EO are available (Rauch et al., 2009) most of them have been developed for use in the organizational context. As regards the instruments used in the educational context, they have generally been validated with university students and have been based either on the three dimensions defined by Covin and Slevin in 1991 (e.g., Taatila and Down, 2012; Mutlutürk and Mardikyan, 2018) or on the five dimensions defined by Lumpkin and Dess, 1996 (e.g., Bolton and Lane, 2011; Vogelsang, 2015; Kurniawan et al., 2019). To date, no instrument based on the seven dimensions defined by Krauss et al. (2005) has been used in the educational field. Therefore, we consider it necessary to develop a new instrument that is based on this theoretical model and which includes the dimensions of achievement orientation and learning orientation. Furthermore, given the controversy surrounding the dimensionality of the construct, a number of authors have pointed out that the development of new instruments could make a considerable contribution to our understanding of EO (Rauch et al., 2009).

The first objective of the present study was therefore to develop a reliable and valid instrument for measuring EO, the *Entrepreneurial Orientation Scale* (EOS), and to examine its psychometric properties. More specifically, we aimed to provide evidence of its internal structure, of measurement invariance across gender groups, and of reliability of scores in terms of both internal consistency and temporal stability. Finally, we also sought to provide evidence of convergent validity.

With the aim of helping to clarify the relationships between EO and other relevant variables, the second objective was to explore latent and observed mean differences across gender and to examine the concurrent relationships of EO with self-efficacy and personal initiative. Given that the study was conducted in the educational field of vocational training, we considered that it would be more appropriate to work with the construct of GSE, rather than ESE, because vocational students do not usually have the immediate intention to start a new business.

## Materials and Methods

### Participants

The sample comprised 411 students (204 female, 207 male) aged between 16 and 57 years (*M* = 22.91; *SD* = 6.26). They were recruited from across 13 vocational training colleges in the Basque Country (Spain), and were enrolled in courses at either the intermediate (17.8% of participants) or advanced (82.2% of participants) level of training. Overall, 53% of the sample had previous work experience, 34.1% had taken part in courses or activities related to entrepreneurship, and 54.3% attended publicly-funded colleges. Sampling was incidental, but in order to ensure that the sample size was sufficient for carrying out the multi-group confirmatory factor analysis (CFA) by gender, we recruited a minimum of 200 participants per group (González-Romá et al., 2006; Pendergast et al., 2017).

### Instruments

#### Entrepreneurial Orientation Scale (EOS)

In a preliminary stage of the present study, we drew up 85 items covering the seven dimensions featured in the aforementioned theoretical model of EO. Sixty-five of these items were positively worded (i.e., stronger agreement with the statement indicated a higher level of EO), while the remainder were negatively worded. This initial battery of items was then submitted to a panel of experts who were asked to rate the relevance of the statements to the construct of EO and to indicate the dimension to which they believed each one corresponded. The panel of experts comprised four university lecturers and three enterprise project coordinators from different institutions. Based on their feedback, we selected items that fulfilled the following two criteria: mean score for relevance above 2.5 (on a scale of 1–4) and matched to the corresponding theoretical dimension by a majority of the experts. This process produced a list of 58 items.

We then piloted this preliminary measure in a sample comprising 82 vocational training students (48% male, 52% female) from three different colleges and four stages of training. Of these students, 34.1% had previous work experience. Analysis of the data obtained – both quantitative (descriptive analysis and corrected item-total correlations) and qualitative (analysis of items that students found difficult to understand) – led us to eliminate 14 items and reformulate a further five. The version of the EOS used in the present study therefore comprised 44 items, each rated on a five-point Likert-like scale (1 = *Totally agree* to 5 = *Totally disagree*). The final version of the instrument contained 32 items. Additional information about the process of developing the instrument can be found in the Supplementary Material (Tables 1, 2).

**Table 1:** Fit indices for the CFA testing the unidimensional and six-factor models.

Models	χ^2^ (*df*)	CFI	TLI	RMSEA (90% CI)
CFA 1 dim. congeneric	2412.123 (464)	0.632	0.606	0.101 (0.097–0.105)
CFA 6 dim. congeneric	875.366 (449)	0.919	0.911	0.048 (0.043–0.053)
CFA 6 dim. tau-equivalent	1172.559 (475)	0.868	0.862	0.060 (0.055–0.064)


*χ^2^, Chi squared; df, degrees of freedom; CFI, comparative fit index; TLI, Tucker-Lewis index; RMSEA, root mean square error of approximation; CI, confidence interval.*

**Table 2:** Standardized factor loadings from the CFA of the six-factor model (*N* = 411).

Items	F1	F2	F3	F4	F5	F6
6. I like teachers with a different approach and who make use of new teaching methods.	0.75					
13. My goal is to have a job that is more about routine than creativity.	0.45					
18. I like to work and take part in groups where new or innovative ideas emerge.	0.72					
25. I like innovative teachers more than traditional ones.	0.72					
1. You have to take risks at times in order to be successful in life.		0.53				
7. I like to make risky decisions.		0.57				
8. In order to create something of value, you have to be prepared to make mistakes.		0.32				
17. I admire people who assume large risks.		0.69				
29. In order to create something of value, you need to take risks.		0.58				
5. I take the initiative whenever I have the opportunity to do so.			0.71			
16. In class I’m often the first person to propose things.			0.65			
27. I like to take the initiative in almost everything I do.			0.64			
2. I usually compete with my classmates.				0.72		
3. For me, being competitive is a good thing.				0.67		
9. Life in general is all about competition.				0.39		
19. I often strive to be better than others.				0.73		
20. I prefer not to have to compete.				0.50		
24. I like teachers who encourage competitiveness among their students.				0.69		
28. I often bet my classmates that I’m better than they are at something.				0.48		
30. I see myself becoming a businessman/woman and always competing.				0.67		
10. Before beginning a task I need to set myself some clear goals.					0.52	
11. Trying to do better (in my studies, in sport, etc.) is important to me.					0.73	
14. I get a special feeling whenever I achieve a goal (in my studies, in sport, etc.).					0.57	
23. I like to set myself goals that imply a challenge (in class, in sport, etc.).					0.73	
31. In order to achieve a goal I usually break it down into smaller objectives.					0.44	
4. My goal is to have a job where I am constantly learning new things.						0.65
12. You learn from your mistakes.						0.47
15. Life is a constant learning process.						0.69
21. I like people who never stop learning.						0.76
22. I try to learn new things every day.						0.72
26. For a company to be successful, its employees have to be learning all the time.						0.55
32. I always try to learn from my experiences.						0.69


*Original items were in Spanish, their English translation is provided.*

#### Entrepreneurial Attitude Scale (Roth and Lacoa, 2009)

This is a unidimensional instrument consisting of 15 items (e.g., “I’m always ready to take on new projects”) that are rated on a four-point Likert-like scale (1 = *Totally disagree* to 4 = *Totally agree*). The statements relate to proactiveness, propensity to excellence, effectiveness seeking, trust in success, and resilience. The instrument shows adequate psychometric properties (Roth and Lacoa, 2009). As this scale was originally developed for application in a Bolivian population, in a previous study small changes were made to three items so as to adapt them to the cultural context of the Basque Country (Balluerka et al., 2014). The scores obtained with this modified instrument yielded an alpha coefficient (internal consistency) of 0.92. The instrument used in the present study had a single factor and an ordinal omega coefficient (internal consistency) of 0.90 (95% CI 0.80–1.00).

#### Spanish Adaptation of the General Self-Efficacy Scale (Baessler and Schwarzer, 1996; Sanjuán et al., 2000)

This instrument assesses perceived personal competence in dealing effectively with a wide variety of stressful situations. It consists of 10 items (e.g., “I can solve most problems if I invest the necessary effort”) that are rated on a ten-point Likert-like scale (1 = *Totally disagree* to 10 = *Totally agree*). The Spanish adaptation shows adequate psychometric properties (Sanjuán et al., 2000). The internal consistency of the score was α = 0.87 and the predictive validity indexes were good. In the present study the internal consistency was good (ordinal omega coefficient = 0.92 [95% CI 0.82–1.00]).

#### Scale for Measuring Personal Initiative in the Educational Field (EMIPAE, Balluerka et al., 2014)

This is a three-factor instrument consisting of 17 items. The factors are proactivity and prosocial behavior (e.g., “I usually participate actively in the classroom/workshop/laboratory, even if I do not receive anything in return”), persistence [e.g., “When I no longer understand the contents of a module/project/subject, I get frustrated and give up” (reverse-scored item)], and self-starting (e.g., “I am particularly good at putting into practice the ideas I had in the classroom/workshop/laboratory”). The items are rated on a five-point Likert-like scale (1 = *Totally disagree* to 5 = *Totally agree*). The instrument shows adequate psychometric properties (Balluerka et al., 2014). Internal consistency indexes (α_proactivity_ = 0.72, α_persistence_ = 0.73, and α_self-starting_ = 0.57) were acceptable and the scores showed evidence of convergent validity and criterion validity. Scores in the present study yielded satisfactory internal consistency indices (omega_proactivity_ = 0.87 [95% CI 0.76–0.96], omega_persistence_ = 0.86 [95% CI 0.78–0.94], and omega_self-starting_ = 0.74 [95% CI 0.63–0.85]).

#### Sociodemographic Data Sheet

This was developed *ad hoc* for the present study in order to collect data on gender, age, the college where students were enrolled, level of studies (intermediate or advanced), course year, previous work experience, and profession (in the case of previous experience).

### Procedure

The 44-item version of the EOS and the instruments required for its validation were administered to participants. The order of administration was as follows: Sociodemographic data sheet, the EOS, the EMIPAE, the Entrepreneurial Attitude Scale, and the GSE Scale. The study was approved by the Research and Teaching Ethics Committee of the University of the Basque Country. In accordance with the Declaration of Helsinki, written informed consent was sought from the heads of the training colleges, from the parents or legal guardians of students who were still minors, and from participants themselves.

### Data Analysis

In order to select the items that would be included in the validated version of the EOS we calculated corrected item-total correlations within each dimension. Items were retained if they achieved a corrected item-total correlation of 0.30 or higher. The criterion for maintaining a dimension was that at least three items yielded a correlation of at least 0.30.

The selected items were then subjected to different models of CFA. The estimator used was weighted least squares mean and variance adjusted (WLSMV), and the fit indices employed were the comparative fit index (CFI) the Tucker-Lewis index (TLI), and the root mean square error of approximation (RMSEA). In the case of the CFI and the TLI, values above 0.90 indicate acceptable fit. For the RMSEA, values below 0.08 indicate acceptable fit and those below 0.06 a good fit (Hu and Bentler, 1999). Factor invariance across gender groups was assessed by means of multi-group confirmatory factor analysis (MG-CFA). The fit indices of the two nested models (the configural invariance model and the scalar invariance model) were compared using the DIFFTEST procedure in order to check that they were not significantly worse in the more restrictive model.

In order to assess the reliability of EOS scores in terms of internal consistency we calculated the ordinal omega coefficient (Gadermann et al., 2012) for each dimension of the instrument; this measure was used as the tau-equivalence required by the alpha coefficient could not be assumed. The temporal stability of EOS scores was evaluated by means of the Spearman rho correlation coefficient. It should be noted that temporal stability was examined in a sub-sample of 65 participants using a 2 weeks interval between test administrations.

In order to obtain evidence of convergent validity we calculated Spearman rho correlation coefficients between the scores obtained by participants on the various dimensions of the EOS and their scores on the Entrepreneurial Attitude Scale (Roth and Lacoa, 2009).

Next, we examined whether there were gender differences in the latent and observed means for each of the dimensions. For the comparison of latent means we constrained the latent mean of the “males” group to 0. Statistical significance was determined on the basis of the *z-*statistic, and the effect size was estimated according to the guidelines proposed by Hancock (2001). In order to test whether the differences in latent means were also found in the observed means we computed observed mean differences (*t*-statistic) and their corresponding effect size (Cohen’s *d*).

Finally, hierarchical multiple regression analyses were performed with the aim of testing the concurrent relationships of EO with GSE and the three dimensions of personal initiative. In these analyses the demographic variables gender, age, and previous work experience were controlled, and thus they were entered in the first step of the regression. In the second step, the demographic variables and all the EO dimensions were entered in the models. In each step, adjusted R squared was calculated. In the second step we also calculated the change in adjusted R squared as a measure of the effect size of the concurrent relationship between EO dimensions and self-efficacy and personal initiative. In addition, zero-order correlations among all variables used in the study were computed. The results can be seen in Supplementary Material (Table 3).

**Table 3:** Fit indices of the models tested to assess measurement invariance across gender groups.

Invariance model	Model constraints	χ^2^ (*df*)	CFI	TLI	RMSEA (90% CI)
M1: Configural invariance	Equivalent model	1353.43 (898)^∗∗∗^	0.911	0.902	0.050 (0.044–0.055)
M2: Metric invariance	Equivalent unstandardized factor loadings	1356.51 (924)^∗∗∗^	0.916	0.910	0.048 (0.042–0.053)
M3: Scalar invariance	Equivalent thresholds	1444.66 (1006)^∗∗∗^	0.915	0.916	0.046 (0.041–0.051)
Differences in model fit (M1–M3)	–	129.01 (108)	–0.004	–	0.004


*χ^2^, Chi squared; df, degrees of freedom; CFI, comparative fit index; TLI, Tucker-Lewis index; RMSEA, root mean square error of approximation; CI, confidence interval. ^∗∗∗^p < 0.001.*

The analyses were performed using SPSS v23 and Mplus v7.4. Missing data (less than 5%) were handled using the single mean imputation procedure.

## Results

### Dimensional Structure

Based on the corrected item-total correlations for the items in each dimension the definitive scale comprised 32 items pertaining to six of the seven dimensions originally proposed: innovativeness, 4 items (e.g., “I like to work and take part in groups where new or innovative ideas emerge”); risk-taking, 5 items (e.g., “In order to create something of value, you need to take risks”); proactiveness, 3 items (e.g., “In class I’m often the first person to propose things”); competitiveness, 8 items (e.g., “I usually compete with my classmates”); achievement orientation, 5 items (e.g., “Before beginning a task I need to set myself some clear goals”); and learning orientation, 7 items (e.g., “My goal is to have a job where I am constantly learning new things”). The autonomy dimension was eliminated as only one of its items had a corrected item-total correlation above the established cut-off.

The unidimensional CFA did not show an adequate fit (see Table 1). However, as can be seen in Table 1 the fit of the six-factor structure was adequate. We also tested a third model in order to determine whether tau-equivalence could be assumed. This model did not show an adequate fit. The factor loadings corresponding to the second (six-factor) model are shown in Table 2. Loadings for all but two of the items were both statistically significant and above 0.40. Observed and latent correlations among the six dimensions can be found in the Supplementary Material (Table 4).

**Table 4:** Reliability indices of the EOS.

Dimension	Mean (*SD*)	Omega (95% CI)	Test-retest correlation
Innovativeness	4.01 (0.63)	0.75 (0.63–0.86)	0.68^∗∗∗^
Risk-taking	3.94 (0.51)	0.68 (0.56–0.80)	0.60^∗∗∗^
Proactiveness	3.37 (0.69)	0.71 (0.59–0.82)	0.64^∗∗∗^
Competitiveness	2.82 (0.71)	0.83 (0.73–0.93)	0.69^∗∗∗^
Achievement orientation	3.89 (0.58)	0.74 (0.60–0.87)	0.63^∗∗∗^
Learning orientation	4.45 (0.42)	0.84 (0.73–0.93)	0.61^∗∗∗^


*^∗∗∗^p < 0.001.*

Table 3 shows the results from the analysis of factor invariance of the EOS across gender groups. The constrained model with equivalent thresholds and factor loadings for males and females (scalar invariance) showed an adequate fit (CFI = 0.915; TLI = 0.916; RMSEA = 0.046), and ΔCFI ≤ 0.01 (0.911–0.915 = -0.004).

### Reliability and Convergent Validity

The ordinal omega coefficients and their confidence intervals are shown in Table 4. These coefficients ranged between 0.68 and 0.84. The test-retest correlation coefficients (Spearman rho) ranged between 0.60 and 0.69 (see Table 4).

The correlation coefficients (Spearman rho) between the participants’ scores on the six dimensions of the EOS and their scores on the Entrepreneurial Attitude Scale were as follows: innovativeness, 0.41; risk-taking, 0.37; proactiveness, 0.56; competitiveness, 0.34; achievement orientation, 0.54; and learning orientation, 0.55 (*p* = 0.001).

### Differences in Entrepreneurial Orientation Across Gender Groups

Having established the scalar invariance of the EOS across gender groups we then compared the means – both latent and observed – obtained by males and females on the six dimensions of the scale. It can be seen in Table 5 that although there were significant differences between males and females on the competitiveness and learning orientation dimensions, the effect sizes for all the comparisons were small.

**Table 5:** Differences between males and females in latent and observed means.

	Latent mean analyses		Observed mean analyses					
			Females		Males			
	*z*	*d*	*M*	*SD*	*M*	*SD*	*t*	*d*
Innovativeness	0.38	0.03	4.02	0.63	4.00	0.62	0.28	0.03
Risk-taking	0.44	0.02	3.95	0.50	3.93	0.53	0.47	0.05
Proactiveness	–0.41	0.02	3.35	0.70	3.38	0.68	–0.42	0.04
Competitiveness	–3.62**	0.22	2.68	0.66	2.96	0.73	–4.06**	0.40
Achievement orientation	–0.32	0.01	3.90	0.57	3.88	0.59	0.33	0.03
Learning orientation	2.93**	0.20	4.52	0.39	4.39	0.44	3.25**	0.32


*^∗∗^p < 0.01.*

### Concurrent Relationships of EO With Self-Efficacy and Personal Initiative

Gender, age, and previous work experience accounted for 1.5% of the variance in self-efficacy. The dimensions of EO accounted for a further 26.5% (large effect size), leading to a total explained variance of 28% (see Table 6). Proactiveness, competitiveness, and learning orientation were significant predictors of self-efficacy. Higher scores on these EO dimensions were related to greater self-efficacy.

**Table 6:** Multiple regressions of control variables and EO dimensions on self-efficacy and personal initiative dimensions.

	Self-efficacy^a^		Proactive and prosocial behavior^b^		Persistence^c^		Self-starting^d^	
				
	β	*t*	β	*t*	β	*t*	β	*t*
**STEP 1**		
Gender (0 = Female; 1 = Male)	0.02	0.44	–0.21	–4.36**	–0.03	–0.63	–0.02	–0.42
Age	–0.01	–0.23	0.09	1.68	0.13	2.40*	0.08	1.49
Work experience (0 = No experience; 1 = Experience)	0.15	2.83**	0.14	2.72**	0.02	0.33	0.12	2.29*
**STEP 2**		
Gender (0 = Female; 1 = Male)	0.03	0.68	–0.18	–4.25**	–0.01	–0.30	–0.02	–0.61
Age	–0.03	–0.63	0.04	0.86	0.10	1.99*	0.06	1.34
Work experience (0 = No experience; 1 = Experience)	0.04	0.79	0.05	1.12	–0.05	–0.91	–0.01	–0.25
Innovativeness	0.08	1.59	0.16	3.42**	0.12	2.31*	0.06	1.25
Risk-taking	–0.02	–0.36	–0.13	–2.76**	–0.17	–3.11**	–0.11	–2.54*
Proactiveness	0.27	5.18**	0.16	3.12**	0.12	2.12*	0.32	6.77**
Competitiveness	0.11	2.30*	0.02	0.49	0.08	1.52	0.16	3.64**
Achievement orientation	0.10	1.89	0.24	4.59**	0.08	1.36	0.22	4.43**
Learning orientation	0.21	3.73**	0.21	3.93**	0.23	3.89**	0.21	4.07**


*^a^R_adj_^2^ = 0.015 for Step 1 (p = 0.027), R_adj_^2^ = 0.280 for Step 2 (p < 0.001), ΔR^2^ = 0.265. ^b^R_adj_^2^ = 0.077 for Step 1 (p < 0.001), R_adj_^2^ = 0.334 for Step 2 (p < 0.001), ΔR^2^ = 0.257. ^c^R_adj_^2^ = 0.013 for Step 1 (p = 0.040), R_adj_^2^ = 0.150 for Step 2 (p < 0.001), ΔR^2^ = 0.137. ^d^R_adj_^2^ = 0.023 for Step 1 (p = 0.006), R_adj_^2^ = 0.405 for Step 2 (p < 0.001), ΔR^2^ = 0.382. ^∗^p < 0.05; ^∗∗^p < 0.01.*

With respect to proactive and prosocial behavior (i.e., the first dimension of personal initiative), gender, age, and work experience explained 7.7% of its variance. An additional 25.7% was explained by the EO dimensions (large effect size), leading to a total explained variance of 33.4% (see Table 6). The only significant demographic predictor was gender, with females scoring higher on proactive and prosocial behavior. All the dimensions of EO, except competitiveness, were significant predictors of this outcome. Specifically, and as indicated by the beta values, higher scores on innovativeness, proactiveness, achievement orientation, and learning orientation were associated with greater proactive and prosocial behavior. Conversely, higher scores on risk-taking were related to lower scores on proactive and prosocial behavior.

The demographic variables explained 1.3% of the variance in persistence. An additional 13.7% was explained by the EO dimensions (medium effect size), leading to a total explained variance of 15% (see Table 6). In addition to age (demographic variable), the EO dimensions of innovativeness, risk-taking, proactiveness, and learning orientation were significant predictors of persistence. Specifically, participants scored higher on persistence with increasing age, innovativeness, proactiveness, and learning orientation. With respect to risk-taking, persistence decreased as scores on this dimension increased.

Finally, gender, age, and work experience explained 2.3% of the variance in self-starting. An additional 38.2% was explained by the EO dimensions (large effect size), leading to a total explained variance of 40.5% (see Table 6). All the dimensions of EO, except innovativeness, were significant predictors of self-starting. The beta values indicate that higher scores on proactiveness, competitiveness, achievement orientation, and learning orientation were associated with a higher self-starting score. Again, an increase in risk-taking was related to a lower score on this dimension of personal initiative.

## Discussion

The first aim of this study was to develop an instrument for assessing entrepreneurial orientation and to examine its psychometric properties in the educational context. The resulting Entrepreneurial Orientation Scale (EOS) comprised 32 items distributed across six dimensions (one of the seven dimensions originally considered, namely autonomy, was eliminated). Given the debate regarding the construct of entrepreneurial orientation we tested both a unidimensional model and a multidimensional (six-factor) model and found that the latter showed the best fit. As to why the autonomy dimension did not function adequately in the educational context, a possible explanation is that, in contrast to the organizational context in which entrepreneurial orientation has traditionally been assessed, autonomy is not an aspect that is widely addressed in the context of our country’s education system. It is worth remembering that in the organizational context, autonomy refers to the independent actions that are implemented by leaders and teams with the aim of launching a new venture (Lumpkin and Dess, 1996). A similar result to ours was obtained in the study by Bolton and Lane (2011), who found that the items designed to measure autonomy did not load on an independent factor, leading them to conclude that autonomy may be a characteristic that, among students, has yet to become consolidated. In a similar vein, Kurniawan et al. (2019) pointed out that the autonomy dimension is not correlated with entrepreneurial intention and therefore it lacks external validity. It should also be noted that other instruments (see, for example, Sánchez, 2010; Bolton and Lane, 2011; Taatila and Down, 2012; Ismail et al., 2015) do not include the achievement orientation and learning orientation dimensions that form part of the EOS, both of which are particularly relevant to the educational setting. Consequently, we believe that the EOS can provide a more comprehensive assessment of entrepreneurial orientation in the academic context.

Importantly, scores on the EOS showed measurement invariance across gender groups, which is a prerequisite for an analysis of differences in mean scores obtained by males and females. The scores also showed adequate reliability in terms of both temporal stability and internal consistency. In addition, the correlations with respect to the Entrepreneurial Attitude Scale may be considered as evidence of good convergent validity. The highest correlation coefficients were those for proactiveness, achievement orientation, and learning orientation, which is what one would expect given that the items of the Entrepreneurial Attitude Scale refer to proactiveness, propensity to excellence, effectiveness seeking, trust in success, and resilience.

The second objective of this study was to explore latent and observed mean differences across gender and to examine the concurrent relationships of EO with self-efficacy and personal initiative. Although gender differences in entrepreneurial orientation have been examined with other instruments, the EOS is the first for which the equivalence of the factor structures, the factor loadings, and the thresholds have been analyzed for males and females. In our study, conducted in the educational context, we found no significant differences between male and female students on four of the six dimensions, and the effect sizes for all the comparisons were small. These results are consistent with those reported by Hunt (2016) for the general construct of entrepreneurial orientation in a sample of undergraduates, as well as with the findings of Pérez-Quintana (2013) and Lim and Envick (2013) with respect to the innovativeness dimension, and with those of Reyes et al. (2014) in relation to risk-taking, once again with samples of university students. These results suggest that the gender differences observed in the organizational context are not present in the same way among students. It should also be noted that, as would be expected due to scalar invariance, we obtained practically the same results when analyzing gender differences using latent and observed scores. This suggests that the EOS has low measurement error and, therefore, that applied researchers may work with observed variables when using the instrument.

Our study, conducted in the educational field, revealed a relationship between EO and self-efficacy, which is consistent with the results obtained by Mohd et al. (2014) in the organizational setting, and by Sesen (2013) with university students. Specifically, we found that the EO dimensions of proactiveness, competitiveness, and learning orientation explained a considerable part of the variance in self-efficacy.

Regarding personal initiative, which is considered one of the eight key competencies for personal development, active citizenship, social inclusion, and employment (European Commission, 2007), EO dimensions showed large concurrent relationships, especially in relation to self-starting. The EO dimensions that predicted all three dimensions of personal initiative were proactiveness, learning orientation, and risk-taking. The negative sign of the relationship between risk-taking and personal initiative was initially surprising, since it indicated that after controlling for demographic variables and the other EO dimensions, a stronger risk-taking orientation was related to less personal initiative. However, an in-depth analysis of the characteristics of the assessment instruments used revealed that the items comprising the risk-taking dimension do not, unlike those for the other dimensions, make reference to the classroom or the educational field, but rather refer more broadly to various aspects of life (see, in Table 2, the content of items 1, 7, 8, 17, and 29). This is important because the instrument used to assess personal initiative refers clearly to the classroom context. At all events, the standardized coefficient of this variable in the explanatory model is the smallest in two of the three dimensions of personal initiative. Finally, it should be noted that the relationship between proactiveness and personal initiative is congruent with studies conducted in organizational settings (Koop et al., 2000; Krauss et al., 2005).

One of the limitations of the present study concerns the sole use of self-report measures, such that the results may be affected by single-method bias. In addition, all the participants came from the same geographical region. Future studies should aim to use other types of measures and to recruit more heterogeneous samples. Another limitation is that we did not test the incremental validity of the EOS in comparison with other published EO measures. This would be an important step in future research with the EOS.

Despite these limitations, we believe that the development and validation of an instrument for assessing, in the educational context, six dimensions of the construct of entrepreneurial orientation makes an important contribution to the field. The results support the multidimensional nature of this construct, which to date has not been examined with vocational training students who will shortly be entering the labor market. A further strength of our study is that we examined measurement invariance across gender groups. The instrument presented here may be used to evaluate initiatives designed to promote an entrepreneurial spirit in schools, colleges, and universities and it therefore provides added value to future research and applications.

## Ethics Statement

This study was carried out in accordance with the recommendations and the ethical standards of the institutional research committee and with the 1964 Helsinki Declaration and its later amendments. The protocol was approved by the Research and Teaching Ethics Committee of the University of the Basque Country. Informed consent was sought from the heads of the training colleges, from the parents or legal guardians of students who were still minors and from participants themselves in accordance with the Declaration of Helsinki.

## Author Contributions

AG, IU, and AM analyzed the theoretical framework of entrepreneurial orientation, designed the study, and wrote the first draft of the manuscript. JA and NB analyzed the data and wrote the Materials and Methods and Results section. GS and AA collected the data. All authors contributed to manuscript revision and proofreading and approved the submitted version of the manuscript.

## Conflict of Interest Statement

The authors declare that the research was conducted in the absence of any commercial or financial relationships that could be construed as a potential conflict of interest.
