# Supplementary material for: Assessment of Entrepreneurial Orientation in Vocational Training Students: Development of a New Scale and Relationships With Self-Efficacy and Personal Initiative
Source: Front Psychol. 2019 May 14;10:1125. doi: 10.3389/fpsyg.2019.01125 (PMC6527836; doi:10.3389/fpsyg.2019.01125)
Supplement: Supplementary file 1 [file Table_1.docx]

**SUPPLEMENTARY MATERIAL**

**Table 1. Procedure of items selection and scale development: Initial items, their assignment to the seven dimensions, positive or negative wording, adjusted item-total correlations in Pilot study, loading in AFE in Main study, selected items in final version**

| **Item number** | **Initial items** | **Dimension** | **Wording** | **Item-total correlation**  **(Pilot study)** | **Item-total correlation (Main study)** | **Selected in final version** | **Number in final version** |
| --- | --- | --- | --- | --- | --- | --- | --- |
| 1 | Admiro a los/as grandes innovadores/as empresariales (Amancio Ortega, Bill Gates, Steve Jobs…). | Innovativeness | Positive |  |  |  |  |
| 2 | Me gusta trabajar y participar en grupos en los que surgen nuevas ideas/ideas innovadoras. | Innovativeness | Positive | .498 | .392 | ✔ | 18 |
| 3 | Sigo la máxima “Innovar o morir”. | Innovativeness | Positive | .384 | .214 |  |  |
| 4 | En general, creo que todo está inventado. | Innovativeness | Negative |  |  |  |  |
| 5 | Me gustan los/as profesores/as que hacen las cosas de manera diferente incorporando nuevos métodos de enseñanza. | Innovativeness | Positive | .485 | .441 | ✔ | 6 |
| 6 | No me gustan las personas que se comportan de manera original y diferente. | Innovativeness | Negative |  |  |  |  |
| 7 | Aspiro a tener un trabajo más rutinario que creativo. | Innovativeness | Negative | .412 | .373 | ✔ | 13 |
| 8 | Prefiero que las clases sean rutinarias, sin sorpresas ni sobresaltos. | Innovativeness | Negative |  |  |  |  |
| 9 | Me gustan más los/as profesores/as innovadores/as que los/as tradicionales. | Innovativeness | Positive | .494 | .522 | ✔ | 25 |
| 10 | A veces, para solucionar un problema, hay que desmarcarse de los procedimientos habituales. | Innovativeness | Positive | .302 | .295 |  |  |
| 11 | Me gusta buscar soluciones a problemas que nadie ha resuelto. | Innovativeness | Positive |  |  |  |  |
| 12 | A veces, para conseguir un determinado objetivo, he hecho cosas que a la gente le han parecido extrañas. | Innovativeness | Positive |  |  |  |  |
| 13 | No me gusta cuestionar las cosas. | Innovativeness | Negative |  |  |  |  |
| 14 | Siento una especie de emoción cuando hago las cosas de manera nueva o diferente. | Innovativeness | Positive |  |  |  |  |
| 15 | En relación a mi futuro laboral, tengo una idea innovadora que espero poder llevar a la práctica. | Innovativeness | Positive | .247 |  |  |  |
| 16 | Admiro a las personas que asumen riesgos importantes. | Risk-taking | Positive | .504 | .571 | ✔ | 17 |
| 17 | Para tener éxito en la vida, a veces hay que arriesgar. | Risk-taking | Positive | .456 | .320 | ✔ | 1 |
| 18 | Me gusta tomar decisiones arriesgadas. | Risk-taking | Positive | .344 | .396 | ✔ | 7 |
| 19 | No me asusta tomar decisiones equivocadas. | Risk-taking | Positive | .371 | .218 |  |  |
| 20 | Prefiero tomar decisiones erróneas que quedarme de brazos cruzados. | Risk-taking | Positive |  |  |  |  |
| 21 | Es normal que la persona que toma las decisiones a veces cometa errores. | Risk-taking | Positive | .429 | .243 |  |  |
| 22 | La gente que arriesga acaba siempre perdiendo. | Risk-taking | Negative | .211 |  |  |  |
| 23 | Las empresas que no arriesgan desaparecen. | Risk-taking | Positive | .420 | .230 |  |  |
| 24 | Cuando tengo que tomar una decisión difícil, me agobio demasiado. | Risk-taking | Negative | .095 |  |  |  |
| 25 | Prefiero que las decisiones difíciles las tomen otros. | Risk-taking | Negative |  |  |  |  |
| 26 | Para crear algo de valor, hay que asumir riesgos. | Risk-taking | Positive | .612 | .449 | ✔ | 29 |
| 27 | Para crear algo de valor, hay que equivocarse previamente. | Risk-taking | Positive | .467 | .302 | ✔ | 8 |
| 28 | Un/a buen/a empresario/a nunca tiene que asumir riesgos. | Risk-taking | Positive | .236 |  |  |  |
| 29 | Me gusta llevar la iniciativa en casi todo lo que hago. | Proactiveness | Positive | .637 | .487 | ✔ | 27 |
| 30 | Tomo la iniciativa en cuanto tengo la oportunidad para ello. | Proactiveness | Positive | .517 | .402 | ✔ | 5 |
| 31 | Un/a buen/a trabajador/a no tiene que tener iniciativa. | Proactiveness | Negative | .377 | .113 |  |  |
| 32 | Para crear una empresa lo que hace falta es dinero, no iniciativa. | Proactiveness | Negative | .017 |  |  |  |
| 33 | A menudo trato de anticiparme a las ideas y acciones de los/as demás. | Proactiveness | Positive | .219 |  |  |  |
| 34 | A menudo soy el/la primero/a en proponer cosas en clase. | Proactiveness | Positive | .581 | .390 | ✔ | 16 |
| 35 | Para que una empresa funcione, debe buscar nuevas oportunidades continuamente. | Proactiveness | Positive |  |  |  |  |
| 36 | Admiro a esas personas que van siempre un paso por delante de las demás. | Proactiveness | Positive |  |  |  |  |
| 37 | Las oportunidades casi nunca vienen solas, hay que buscarlas. | Proactiveness | Positive | .400 | .185 |  |  |
| 38 | Me considero una persona independiente. | Autonomy | Positive | .328 | .218 |  |  |
| 39 | Yo soluciono mis propios problemas. | Autonomy | Positive | .106 |  |  |  |
| 40 | Yo no necesito la aprobación de los/as demás para hacer lo que debo. | Autonomy | Positive | .335 | .314 |  |  |
| 41 | Es importante ser uno/a mismo/a y no depender de lo que digan los demás. | Autonomy | Positive | .219 |  |  |  |
| 42 | Para mí, el mayor reconocimiento es el que me concedo a mí mismo/a. | Autonomy | Positive | .322 | .122 |  |  |
| 43 | No me gustan las personas que dependen demasiado de los/as demás. | Autonomy | Positive | .180 |  |  |  |
| 44 | Aspiro a tener un trabajo en el que me limite a cumplir órdenes. | Autonomy | Negative |  |  |  |  |
| 45 | Me importa poco lo que los/as demás puedan pensar de mí. | Autonomy | Positive | .400 | .249 |  |  |
| 46 | Me gustaría ser mi propio/a jefe/a. | Autonomy | Positive | .173 |  |  |  |
| 47 | En general, las personas deberían ser más independientes | Autonomy | Positive |  |  |  |  |
| 48 | A menudo me esfuerzo al máximo para superar a los/as demás. | Competitiveness | Positive | .676 | .579 | ✔ | 19 |
| 49 | Me gusta competir con mis compañeros/as de clase. | Competitiveness | Positive | .632 | .591 | ✔ | 2 |
| 50 | Cuando compito (por ejemplo en algún deporte) suelo ser bastante agresivo/a. | Competitiveness | Positive |  |  |  |  |
| 51 | Me gusta desafiar a otros compañeros/as de clase. | Competitiveness | Positive |  |  |  |  |
| 52 | A menudo apuesto con mis compañeros/as a que soy mejor que ellos/as en algo. | Competitiveness | Positive | .405 | .445 | ✔ | 28 |
| 53 | Me gustan los/as profesores/as que fomentan la competitividad entre alumnos/as. | Competitiveness | Positive | .583 | .613 | ✔ | 24 |
| 54 | No me gustan las actividades en las que tengo que competir con mis compañeros/as. | Competitiveness | Negative |  |  |  |  |
| 55 | La vida, en general, es pura competición. | Competitiveness | Positive | .452 | .318 | ✔ | 9 |
| 56 | Admiro a esos/as ejecutivos/as de empresa que compiten como tiburones. | Competitiveness | Positive |  |  |  |  |
| 57 | En el futuro me veo como empresario/a, siempre compitiendo. | Competitiveness | Positive | .643 | .504 | ✔ | 30 |
| 58 | Para mí ser competitivo/a es una virtud. | Competitiveness | Positive | .659 | .570 | ✔ | 3 |
| 59 | Prefiero no tener que competir que tener que hacerlo. | Competitiveness | Negative | .444 | .429 | ✔ | 20 |
| 60 | En los retos y desafíos suelo dar lo mejor de mí mismo/a. | Competitiveness | Positive |  |  |  |  |
| 61 | Detesto los retos y desafíos. | Competitiveness |  |  |  |  |  |
| 62 | Antes de comenzar con alguna tarea, necesito marcarme claramente los objetivos. | Achievement orientation | Positive | .547 | .423 | ✔ | 10 |
| 63 | Me gusta marcarme objetivos que supongan un reto (en la escuela, en el deporte…). | Achievement orientation | Positive | .548 | .483 | ✔ | 23 |
| 64 | Conseguir o no conseguir mis objetivos (en la escuela, en el deporte…) no depende de mí. | Achievement orientation | Negative |  |  |  |  |
| 65 | Yo soy el/la principal responsable de los resultados que obtengo (en la escuela, en el deporte…). | Achievement orientation | Positive | .065 |  |  |  |
| 66 | Cuando persigo el mismo objetivo que otros/as compañeros/as, suelo compararme con ellos/as para saber si lo voy alcanzando. | Achievement orientation | Positive |  |  |  |  |
| 67 | Siento una emoción especial cuando alcanzo un objetivo (en la escuela, en el deporte…). | Achievement orientation | Positive | .364 | .360 | ✔ | 14 |
| 68 | Normalmente empiezo a hacer las cosas sin planificarlas (en la escuela, en el deporte…). | Achievement orientation | Negative | .278 |  |  |  |
| 69 | Para conseguir un gran objetivo, suelo dividirlo en varios objetivos más pequeños. | Achievement orientation | Positive | .349 | .364 | ✔ | 31 |
| 70 | A veces preferiría hacer otras cosas pero me sacrifico y hago lo que debo. | Achievement orientation | Negative |  |  |  |  |
| 71 | Admiro a las personas que se sacrifican para alcanzar aquello que desean. | Achievement orientation | Positive | .338 | .286 |  |  |
| 72 | Para mí, es importante tratar de superarme a mí mismo/a (en la escuela, en el deporte…). | Achievement orientation | Positive | .641 | .537 | ✔ | 11 |
| 73 | El/La que no comete errores no aprende. | Learning orientation | Positive | .258 |  |  |  |
| 74 | No se puede aprender nada valioso sin cometer errores. | Learning orientation | Positive |  |  |  |  |
| 75 | Aspiro a tener un trabajo en el que pueda aprender cosas nuevas constantemente. | Learning orientation | Positive | .515 | .483 | ✔ | 4 |
| 76 | Me gusta la gente que nunca deja de aprender. | Learning orientation | Positive | .439 | .524 | ✔ | 21 |
| 77 | Para que una empresa vaya bien, sus trabajadores/as tienen que estar aprendiendo constantemente. | Learning orientation | Positive | .535 | .364 | ✔ | 26 |
| 78 | Me basta con saber lo justo, no pienso perder el tiempo aprendiendo cosas innecesarias. | Learning orientation | Negative |  |  |  |  |
| 79 | Estudiar sirve sobre todo para conseguir un trabajo. | Learning orientation | Negative |  |  |  |  |
| 80 | Siempre trato de aprender de las experiencias. | Learning orientation | Positive | .439 | .515 | ✔ | 32 |
| 81 | Me da vergüenza cometer errores en clase. | Learning orientation | Negative |  |  |  |  |
| 82 | De los errores se aprende. | Learning orientation | Positive | .331 | .324 | ✔ | 12 |
| 83 | Trato de aprender cosas nuevas cada día. | Learning orientation | Positive | .583 | .553 | ✔ | 22 |
| 84 | La vida es un aprendizaje constante. | Learning orientation | Positive | .358 | .452 | ✔ | 15 |
| 85 | Cuando termine la escuela, pienso seguir estudiando por mi cuenta. | Learning orientation | Positive | .203 |  |  |  |

Note: Adjusted item-total correlations below .30 are shown in red.

**Table 2. Reworded items in pilot study**

| **Item number** | **Initial item** | **Reworded item** |
| --- | --- | --- |
| 19 | No me asusta tomar decisiones equivocadas | No me asusta equivocarme cuando toma una decisión |
| 40 | Yo no necesito la aprobación de los/as demás para hacer lo que debo | Yo no necesito la aprobación de los/as demás para desarrollar mis ideas |
| 45 | Me importa poco lo que los/as demás puedan pensar de mí | Me importa poco lo que los/as demás puedan pensar de mis ideas |
| 53 | Me gustan los/as profesores/as que fomentan la competitividad entre alumnos/as | Me gustan los/as profesores/as que impulsan la competitividad entre alumnos/as |
| 59 | Prefiero no tener que competir que tener que hacerlo | Prefiero no tener que competir |

**Table 3. Zero-order correlations among all variables used in the study**

|  | **Entrepreneurial Orientation** | | | | | |
| --- | --- | --- | --- | --- | --- | --- |
|  | **Innovativeness** | **Risk-taking** | **Proactiveness** | **Competitiveness** | **Achievement orientation** | **Learning orientation** |
| **AES** | .41*** | .37*** | .56*** | .34*** | .54*** | .55*** |
| **Self-efficacy** | .26*** | .26*** | .43*** | .27*** | .37*** | .37*** |
| **Self-starting** | .27*** | .22*** | .52*** | .31*** | .46*** | .40*** |
| **Proactive and prosocial behavior** | .39*** | .15*** | .35*** | .07 | .39*** | .45*** |
| **Persistence** | .26*** | .05 | .23*** | .12* | .22*** | .32*** |

**p* < .05 ****p*<.001

**Table 4. Observed and latent correlations among the six EOS dimensions**

|  | **Innovativeness** | **Risk-taking** | **Proactiveness** | **Competitiveness** | **Achievement orientation** | **Learning orientation** |
| --- | --- | --- | --- | --- | --- | --- |
| **Innovativeness** |  | .51*** | .51*** | .04 | .39*** | .65*** |
| **Risk-taking** | .31*** |  | .64*** | .37*** | .69*** | .59*** |
| **Proactiveness** | .31*** | .37*** |  | .47*** | .70*** | .54*** |
| **Competitiveness** | .02 | .25*** | .32*** |  | .50*** | .09 |
| **Achievement orientation** | .27*** | .46*** | .44*** | .34*** |  | .70*** |
| **Learning orientation** | .48*** | .40*** | .34*** | .05 | .48*** |  |

Note: Latent correlations are shown above the diagonal. Observed correlations are shown below the diagonal.

**p* < .05 ****p*<.001
